# Vocal music enhances memory and language recovery after stroke: pooled results from two RCTs

**DOI:** 10.1002/acn3.51217

**Published:** 2020-10-06

**Authors:** Aleksi J. Sihvonen, Vera Leo, Pablo Ripollés, Terhi Lehtovaara, Aki Ylönen, Pekka Rajanaro, Sari Laitinen, Anita Forsblom, Jani Saunavaara, Taina Autti, Matti Laine, Antoni Rodríguez‐Fornells, Mari Tervaniemi, Seppo Soinila, Teppo Särkämö

**Affiliations:** ^1^ Cognitive Brain Research Unit Department of Psychology and Logopedics Faculty of Medicine University of Helsinki Helsinki Finland; ^2^ Department of Neurosciences Faculty of Medicine University of Helsinki Helsinki Finland; ^3^ Department of Psychology New York University New York New York; ^4^ Private Music Therapy Practitioner Turku Finland; ^5^ Private Music Therapy Practitioner Helsinki Finland; ^6^ Department of Medical Physics Turku University Hospital Turku Finland; ^7^ Department of Radiology HUS Medical Imaging Center Helsinki University Central Hospital University of Helsinki Helsinki Finland; ^8^ Department of Psychology Åbo Akademi University Turku Finland; ^9^ Cognition and Brain Plasticity Group Bellvitge Biomedical Research Institute L’Hospitalet de Llobregat Barcelona Spain; ^10^ Department of Cognition Development and Education Psychology University of Barcelona Barcelona Spain; ^11^ Institució Catalana de Recerca i Estudis Avançats Barcelona Spain; ^12^ CICERO Learning University of Helsinki Helsinki Finland; ^13^ Division of Clinical Neurosciences Department of Neurology Turku University Hospital and University of Turku Turku Finland

## Abstract

**Objective:**

Previous studies suggest that daily music listening can aid stroke recovery, but little is known about the stimulus‐dependent and neural mechanisms driving this effect. Building on neuroimaging evidence that vocal music engages extensive and bilateral networks in the brain, we sought to determine if it would be more effective for enhancing cognitive and language recovery and neuroplasticity than instrumental music or speech after stroke.

**Methods:**

Using data pooled from two single‐blind randomized controlled trials in stroke patients (*N* = 83), we compared the effects of daily listening to self‐selected vocal music, instrumental music, and audiobooks during the first 3 poststroke months. Outcome measures comprised neuropsychological tests of verbal memory (primary outcome), language, and attention and a mood questionnaire performed at acute, 3‐month, and 6‐month stages and structural and functional MRI at acute and 6‐month stages.

**Results:**

Listening to vocal music enhanced verbal memory recovery more than instrumental music or audiobooks and language recovery more than audiobooks, especially in aphasic patients. Voxel‐based morphometry and resting‐state and task‐based fMRI results showed that vocal music listening selectively increased gray matter volume in left temporal areas and functional connectivity in the default mode network.

**Interpretation:**

Vocal music listening is an effective and easily applicable tool to support cognitive recovery after stroke as well as to enhance early language recovery in aphasia. The rehabilitative effects of vocal music are driven by both structural and functional plasticity changes in temporoparietal networks crucial for emotional processing, language, and memory.

## Introduction

During the last decade, there has been growing interest toward music as a neurorehabilitation tool, especially for stroke.^1^ This has been fueled by (1) the rapidly increasing prevalence of stroke and its massive socioeconomic burden and growing need for cost‐effective rehabilitation tools^2^ and (2) advances in music neuroscience, uncovering the wide‐spread cortical and subcortical networks underlying the auditory, motor, cognitive, and emotional processing of music^3^, ^4^ and their malleability by musical training.^5^ In the rehabilitation context, music can be viewed as a form of environmental enrichment (EE) that increases activity‐dependent neuroplasticity in the large‐scale brain network it stimulates.^6^ In animals, EE is a powerful driver of synaptic plasticity, neurotrophin production, and neurogenesis, improving also cognitive‐motor recovery.^7^ In stroke patients, EE where patients are provided additional social interaction and stimulating activities (e.g., games) is emerging as a promising way to increase physical, social, and cognitive activity.^8^

Previously, we explored the long‐term efficacy of musical EE in a three‐arm randomized controlled trial (RCT) comparing daily music listening to a control intervention (audiobook listening) and standard care (SC) in stroke patients. Music listening enhanced the recovery of verbal memory and attention and reduced negative mood^9^ as well as increased gray matter volume (GMV) in spared prefrontal and limbic areas in left hemisphere‐lesioned patients.^10^ Corroborating results were recently obtained in another RCT where daily music listening, alone or in combination with mindfulness training, enhanced verbal memory and attention more than audiobooks.^11^ While these results imply that music listening can be cognitively, emotionally, and neurally effective after stroke, its tailored, more optimized use in stroke rehabilitation requires determining which components of music are specifically driving these effects and which patients benefit most from it.

The vocal (sung) component of music could be one key factor contributing to its rehabilitative efficacy. Singing is one of the oldest forms of human communication, a likely precursor to language evolution.^12^ Songs represent an important interface between speech and music, binding lyrics and melody into a unified representation and engaging linguistic and vocal‐motor brain processes in addition to the auditory, cognitive, and emotional processing associated with instrumental music. fMRI evidence indicates that listening to sung music activates temporal, frontal, and limbic areas more bilaterally and extensively than listening to speech^13^, ^14^ or instrumental music,^15^, ^16^ also in the early poststroke stage.^17^ After unilateral stroke, spared brain regions in both ipsi‐ and contralesional hemisphere undergo spontaneous neuroplasticity changes and steer the recovery of behavioral functions, including speech.^18^ In this regard, the large‐scale bilateral activation induced by vocal music could make it more effective than speech or instrumental music that engage primarily the left or right hemisphere, respectively.^19^

Vocal music is particularly interesting in the domain of aphasia rehabilitation. In nonfluent aphasia, the ability to retain the ability to produce words through singing is often preserved, and aphasic patients are also able to learn new verbal material when utilizing a sung auditory model.^20^ Singing‐based speech training interventions, such as melodic intonation therapy (MIT), have been found effective in enhancing the production of trained speech content and the recovery of verbal communication in aphasia, especially when provided at the subacute poststroke stage.^21^, ^22^ Whether regular listening to vocal music could have long‐term positive effects on early language recovery in aphasia is currently unknown.

In the present study, we use data pooled from two RCTs (*N* = 83), including our previous trial^9^, ^10^ (*N* = 38) and a new, previously unpublished trial (*N* = 45), to (1) determine the contribution of sung lyrics on the cognitive, linguistic, and emotional efficacy of music by comparing daily listening to vocal music, instrumental music, and audiobooks and (2) uncover the structural neuroplasticity (GMV) and functional connectivity (FC) changes underlying them. We hypothesized that (i) vocal music would be superior to instrumental music and audiobooks in enhancing cognitive and language recovery, (ii) both vocal and instrumental music would enhance mood more than audiobooks, and (iii) the rehabilitative effects of vocal music would be linked to GMV changes in temporal, frontal, and parietal regions associated with the processing of language, music, and memory^13^, ^14^, ^15^, ^16^, ^17^ and commonly induced by musical training^5^ as well as increased resting‐state functional connectivity (FC), particularly in the default mode network (DMN),^23^ which has recently been linked to stroke recovery.^24^, ^25^ Moreover, given previous evidence on singing‐based speech rehabilitation in aphasia,^21^, ^22^ we (3) explore whether listening to vocal music can be effective for aphasia recovery.

## Methods

### Subjects and study design

**Figure 1:**
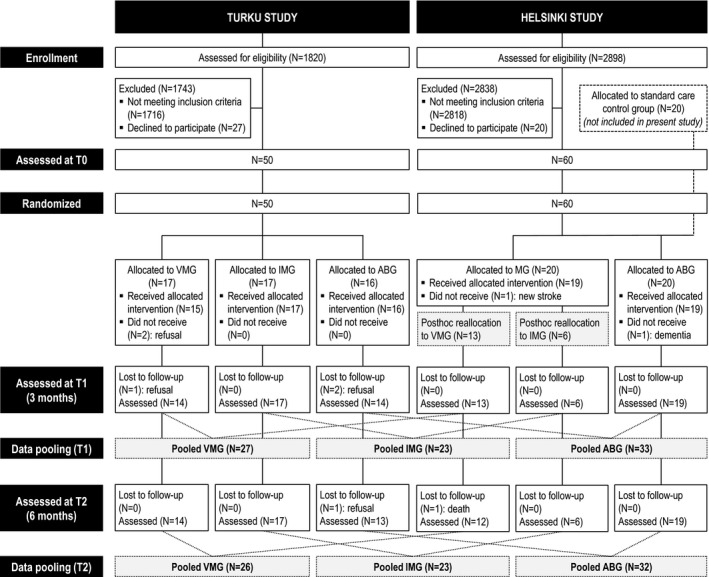
Flow chart outlining the design and progress of the study.

Subjects were stroke patients pooled from two RCTs performed in Turku and Helsinki, Finland. Data pooling was done to increase sample size and statistical power, and was feasible because both studies had common (1) inclusion/exclusion criteria (MRI‐verified acute unilateral stroke; right‐handed; <80 years old; Finnish speaking; able to co‐operate; no hearing loss, prior neurological/psychiatric disease, or substance abuse); (2) assessment time points [<3 weeks (baseline, T0), 3‐month (T1), and 6‐month (T2) poststroke]; (3) outcome measures (see below); and (4) timing, frequency, and delivery of the interventions (see below). No formal power calculations were conducted in the planning of the two studies. The studies were approved by the Ethics committees of the Hospital Districts of Southwest Finland (Turku) and Helsinki and Uusimaa (Helsinki). All patients signed an informed consent and received standard medical treatment and rehabilitation for stroke. Study design and participant flow is shown in Figure 1. In both studies, randomization was stratified for lesion laterality (left/right) and performed as block randomization (10 blocks of three consecutive patients for left and right lesions), the order within the blocks drawn using a random number generator. The randomization list was generated by a laboratory engineer who was not involved in the data collection, and the persons who performed the patient recruitment had no access to it (allocation concealment).

In the Turku RCT (ClinicalTrials.gov identifier: NCT01749709, previously unreported trial), 50 stroke patients (83% of planned sample size of *N* = 60) were recruited during 2013–2016 from the Department of Clinical Neurosciences of Turku University Hospital. The patients were randomized to the following three groups: vocal music group (VMG, *N* = 17), instrumental music group (IMG, *N* = 17), and audiobook group (ABG, *N* = 16). Forty‐five patients completed the trial up to T1 and 44 up to T2. In the Helsinki RCT [nonregistered (data collection began before the ICMJE trial registration recommendations), previously reported^9^ trial], 60 stroke patients (100% of planned sample size) were recruited during 2004–2006 from the Department of Neurology of Helsinki University Central Hospital (HUCH). The patients were randomized to the following three groups (*N* = 20 in each): music group (MG), ABG, and control (SC only) group. In the Helsinki RCT, 55 patients completed the trial up to T1 and 54 up to T2. Those Helsinki MG (*N* = 19) and ABG (*N* = 19) patients who had T1 follow‐up data were included in the present study. Based on the proportion of listening to vocal and instrumental music (data obtained from the listening diaries of the patients and the case notes of the music therapists), the 19 MG patients were reclassified to VMG [*N* = 13; vocal music: mean 82.6% (SD 11.9%)] and IMG (*N* = 6; instrumental music: mean 54.2% (SD 13.3%)], which differed highly significantly regarding the music type [*t*(17) = 6.63, *P* < 0.001]. In other words, the MG patients in the Helsinki study were not randomized to VMG and IMG, but were reallocated to these groups post hoc based on the type of music they had listened to during the intervention period. This was done in order to pool the data with the Turku study. The final pooled data comprised 83 patients from T0 to T1 (VMG: *N* = 27, IMG: *N* = 23, ABG: *N* = 33) and 81 patients from T0 to T2 (VMG: *N* = 26, IMG: *N* = 23, ABG: *N* = 32).

### Intervention

After baseline (T0) assessments, a professional music therapist contacted the patients, informed them of the group allocation, and interviewed them about prestroke leisure activities, including music listening and reading. The therapist provided the patients with a portable player, over‐ear headphones, and a collection of listening material, which was vocal music with sung lyrics in VMG, instrumental music (with no sung lyrics) in IMG, and narrated audiobooks (with no music) in ABG. All material was in a language that the patients understood well (mostly Finnish or English). Within each group, the material was selected individually to match the music/literature preferences of the patient as closely as possible. The music material comprised primarily pop, rock, and schlager music songs in the VMG and classical, jazz, and film score music in the IMG. The patients were trained in using the players and instructed to listen to the material by themselves daily (min. 1 h per day) for the following 2 months at the hospital or at home. They were also asked to keep a listening diary. During the 2‐month intervention, the therapist kept regular contact with the patients to encourage listening, provide more material, and help with the equipment if needed. On average, the therapist spent around 4 h with each participant during the intervention period. After the intervention period, the patients were free to continue listening at their own will.

### Behavioral outcome measures

Neuropsychological testing was performed three times (T0/T1/T2), blinded to the group allocation of the patients. The assessment battery (Table 1) used in both the Turku and Helsinki studies covered three cognitive domains (verbal memory, language skills, and focused attention) and mood (POMS). The raw test scores measuring each domain were added up and these summary scores were used in the statistical analyses to reduce the number of variables.^9^ The primary outcome was verbal memory (change from T0 to T1). The secondary outcomes were verbal memory (change from T0 to T2) as well as language and focused attention (change from T0 to T1 and T2) and POMS scales (at T1 and T2). Parallel versions of memory tests were used in different testing occasions to minimize practice effects. Testing was carried out in a quiet room reserved for neuropsychological studies, over multiple sessions if needed to avoid fatigue. The level of aphasia at T0 was assessed with the BDAE‐ASRS, with score ≤ 4 indicating aphasia. Scoring was done clinically primarily based on conversational speech, drawing information also from the language tests (Table 1). All the aphasic patients had left hemisphere lesions.

**Table 1:** Behavioral measures.

Time	Domain	Measure	Task description
T0	Aphasia	BDAE/ASRS	6‐point rating scale of overall aphasia severity
	Amusia	MBEA/Scale & Rhythm subtests	Compare two consecutive melodies (same/different)
	Music reward	BMRQ (only Turku patients)	20 questions about role of music in life before stroke
T0, T1, T2	Verbal memory	RBMT/Story recall subtest	Remember a short story (immediate and delayed recall)
		Auditory‐verbal learning task	Remember a list of 10 words (three learning trials and delayed recall)
T0, T1, T2	Language skills	Verbal fluency task	List words beginning with a specific letter (60 sec)
		Naming test (CERAD/BNT)	Name items from line drawings
		short Token Test	Follow verbal instructions
T0, T1, T2	Focused attention	CS/Stroop subtest	Name colors of words on screen (congruent/incongruent)
		CS/Mental subtraction subtest	Subtract number on screen from 9
T0, T1, T2	Mood	POMS	Rate current mood (38 items, 8 scales: Tension, Depression, Irritability, Vigor, Fatigue, Inertia, Confusion, Forgetfulness)

Measure abbreviations: ASRS, Aphasia Severity Rating Scale; BDAE, Boston Diagnostic Aphasia Examination; BMRQ, Barcelona Music Reward Questionnaire; BNT, Boston Naming Test; CERAD, Consortium to Establish a Registry for Alzheimer's Disease; CS, CogniSpeed© test battery; MBEA, Montreal Battery of Evaluation of Amusia; POMS, Profile of Mood States; RBMT, Rivermead Behavioural Memory Test.

### Statistical analysis of behavioral data

Demographic and clinical characteristics were analyzed with univariate ANOVAs or nonparametric Kruskal–Wallis tests, and chi‐squared tests. Longitudinal cognitive data were analyzed using mixed‐model ANOVAs with Time as within‐subject factor and Group (VMG/IMG/ABG) and Aphasia as between‐subject factors. Separate mixed‐model ANOVAs were performed to determine the short‐term (Time: T0/T1) and long‐term (Time: T0/T2) effects of the intervention. Due to the emotional lability of the patients at the acute stage, the POMS data were analyzed cross‐sectionally at each time point using univariate ANOVAs. In order to control for the potential effects of the two trial sites (Turku/ Helsinki) and those demographic and clinical variables, in which there were group differences (see Results), in the analyses of outcome measures, trial site, amusia, prestroke music listening, and cross‐listening were included as covariates in the ANOVAs. Post hoc tests of change scores (T1‐T0, T2‐T0) were performed using the Bonferroni correction. All statistical analyses were performed with IBM SPSS Statistics 24. Missing values in the data were considered missing at random and were not replaced.

### MRI data acquisition

Structural MRI was acquired from 75 patients at T0 and T2 using a 1.5T Siemens Vision scanner of the HUCH Department of Radiology (Helsinki, *N* = 33) and a 3T Siemens Verio scanner of the Medical Imaging Centre of Southwest Finland (Turku, *N* = 42). High‐resolution T1 images (Helsinki: flip angle 15°, TR 1900 msec, TE 3.68 ms, voxel size 1.0 × 1.0 × 1.0 mm; Turku: flip angle 9°, TR 2300 msec, TE 2.98 msec, voxel size 1.0 × 1.0 × 1.0 mm) were obtained, coupled with fluid‐attenuated inversion recovery (FLAIR) images used in localizing the lesions. Functional MRI was acquired from 35 Turku patients at T0 and T2 using a single‐shot T2*‐weighted gradient‐echo EPI sequence (flip angle 80°, TR 2010 msec, TE 30 msec, voxel size 2.8 × 2.8 × 3.5 mm, slice thickness 3.5 mm, 32 slices), with a total of 280 functional volumes being collected. The session included a 5‐minute eyes‐open resting‐state condition (rs‐fMRI) and a passive listening task (task‐fMRI) during which auditory stimuli were presented through MR‐compatible headphones using Presentation software (Neurobehavioral Systems, version 16.3). In task‐fMRI, we used a block design where the patients were presented 15‐second excerpts of well‐known Finnish songs with (1) sung lyrics (Vocal, six blocks) and (2) without sung lyrics (Instrumental, six blocks), (3) well‐known Finnish poems (Speech, six blocks), and (4) no auditory stimuli (Rest, 18 blocks). The order of the auditory blocks was randomized across subjects and time, and the rest blocks were presented in between the auditory blocks.

### MRI data preprocessing

MRI data were preprocessed using Statistical Parametric Mapping software (SPM8, Wellcome Department of Cognitive Neurology, UCL) under MATLAB 8.4.0. The structural T1 images of each subject were reoriented to the anterior commissure and then processed using Unified Segmentation^26^ with medium regularization. Lesioned areas were not excluded from subsequent analyses (see below), but cost function masking (CFM)^27^ was applied to achieve accurate segmentation and optimal normalization of the lesioned GM and white matter (WM) tissue, with no postregistration lesion shrinkage or out‐of‐brain distortion. Using MRIcron (https://www.nitrc.org/projects/mricron), CFM was performed by manually depicting the lesioned areas slice‐by‐slice to the T1 images of each subject. The segmented GM and WM images were modulated to preserve the original signal strength and then normalized to the MNI space. After this, to reduce residual interindividual variability, GM and WM probability maps were smoothed using an isotropic spatial filter (FWHM 6 mm). For fMRI data, the functional runs were first realigned and their mean image was calculated. The T1 image and its lesion mask were then co‐registered to this mean functional image. The normalization parameters were again estimated using Unified Segmentation with CFM and were applied to the whole functional run to register it to MNI space. In this registration step, data were resampled into 2.0 × 2.0 × 2.0 mm voxel size. Finally, the normalized fMRI data were smoothed using an 8‐mm FWHM kernel.

### Voxel‐based morphometry

VBM analysis^28^ was performed using SPM8. The individual preprocessed GM and WM images were submitted to second‐level flexible factorial analyses with Time (T0/T2) and Group (VMG/IMG/ABG) as factors, and scanner type (i.e., trial site), age, sex, and total intracranial volume as additional covariates.^29^ Thus, altogether three Group (VMG > ABG, IMG > ABG, and VMG > IMG) × Time (T2 > T0) interactions were calculated. Separate analyses were also performed within the aphasic and non‐aphasic patients. All results were thresholded at an uncorrected *P* < 0.005 threshold at the voxel level, and standard SPM family‐wise error rate (FWE) cluster‐level correction based on random field theory (RFT) with a *P* < 0.05 was used. Only clusters surviving FWE‐corrected *P* < 0.05 at cluster level are reported. For each significant comparison, the minimum size for a cluster to be corrected for multiple corrections (FWEc) is reported. Neuroanatomical areas were identified using the Automated Anatomical Labeling Atlas provided within the xjView toolbox (http://www.alivelearn.net/xjview/).

### Functional connectivity

Group‐level spatial independent component analysis was performed using the Group ICA of fMRI Toolbox (GIFT) software (http://mialab.mrn.org/software/gift/). The ICA spatial components were extracted from the rs‐fMRI and task‐fMRI runs. After performing intensity normalization of the preprocessed fMRI images, data were concatenated and, following previous studies,^30^ reduced to 20 temporal dimensions using principal component analysis and then analyzed using the Infomax algorithm.^31^ From the ICA spatial components representing the different networks, the default mode network (DMN)^23^ was identified and selected for further analyses based on the pattern of VBM results (see Results section). In the rs‐fMRI, to obtain whole‐brain group‐wise statistics, the spatial maps of the DMNs from all patients were submitted to a second‐level flexible factorial analyses with Time (T0/T2) and Group (VMG/IMG/ABG) as factors. In the task‐fMRI, the time course of the DMN was fitted to an SPM model that included the Vocal, Instrumental, and Speech conditions as regressors, yielding beta values representing the engagement of DMN during each condition. These were then analyzed with SPSS using mixed‐model ANOVAs with Time (T0/T2) and Group (VMG/IMG/ABG) as factors. BDAE‐ASRS and BMRQ scores were included as additional covariates. Statistical maps were thresholded at a voxel‐level uncorrected *P* < 0.005 threshold and standard SPM FWE cluster‐level correction based on RFT with a *P* < 0.05 was used. For each significant comparison, the minimum size for a cluster to be corrected for multiple corrections (FWEc) is reported. Finally, in order to determine the link between the behavioral outcome and the VBM‐FC results, correlation analyses (Pearson, two‐tailed, FDR‐corrected) were performed between changes (T1‐T0, T2‐T0) in language and verbal memory and the clusters showing volume or FC changes between the groups.

## Results

The participant flow in the two studies is presented in Figure 1. Overall, adherence was very good and similar between the trial sites, with 83 of 90 (92%) patients (Turku: 90%, Helsinki: 95%) completing the study up to the 3‐month stage (T1) and 81 of 90 (90%) patients (Turku: 88%, Helsinki: 93%) up to the 6‐month stage (T2). The pooled data from the Turku and Helsinki studies were analyzed to determine the short‐term (from T0 to T1) and long‐term (from T0 to T2) effects of the music intervention.

### Group comparability at baseline

At T0, there were no statistically significant differences between VMG, IMG, and ABG in most demographic and clinical characteristics and prestroke leisure activities (Table 2). Prestroke music listening frequency showed a group difference [Kruskal–Wallis *H* = 11.81, *P* = 0.003], with more prestroke music listening in IMG than in VMG (*P* = 0.007) or ABG (*P* = 0.010). Also the proportion of amusic patients differed between the three groups [χ^2^(2) = 9.29, *P* = 0.010], with less amusics in IMG than in VMG (*P* = 0.034) or ABG (*P* = 0.001). These baseline differences were considered to be due to chance as there is no reason to assume any systematic bias between the groups. The groups were comparable in the behavioral outcome measures at T0 (Tables 3 and 4).

**Table 2:** Baseline demographic and clinical characteristics and prestroke leisure activities of the patients.

	VMG *N* = 27	IMG *N* = 23	ABG *N* = 33	*P*‐value
Demographic characteristics				
Age (years)	54.9 (13.4)	56.7 (10.3)	59.8 (11.6)	0.267 (F)
Gender (male/female)	15/12	15/8	16/17	0.464 (χ^2^)
Education (years)	12.8 (4.5)	13.5 (3.8)	12.1 (3.5)	0.405 (F)
Prestroke leisure activities^a^				
Music listening	4.2 (1.2)	4.7 (0.9)	3.6 (1.5)	**0.003 (H)**
Radio listening	3.3 (1.9)	3.4 (1.4)	3.8 (1.4)	0.545 (H)
Reading	3.9 (1.7)	4.3 (1.5)	4.6 (0.8)	0.138 (H)
Clinical characteristics				
Time from stroke to T0 (days)	6.1 (2.6)	7.2 (5.0)	7.3 (4.1)	0.502 (F)
Lesion laterality (left/right)	15/12	10/13	16/17	0.690 (χ^2^)
Lesion size (cm^3^)	46.8 (50.8)	51.4 (50.9)	45.1 (45.5)	0.893 (F)
Aphasia (yes/no)^b^	10/17	5/18	14/19	0.269 (χ^2^)
Aphasia severity^b^	3.1 (1.3)	3.6 (0.9)	3.2 (1.1)	0.593 (H)
Amusia (yes/no)^c^	19/7	8/15	23/10	**0.010 (χ^2^)**

Data are mean (SD) unless otherwise stated. Significant group differences are shown in bold. Abbreviations: ABG, Audiobook group; F, one‐way ANOVA; H, Kruskal–Wallis test; IMG, Instrumental music group; T0, baseline (acute); VMG, Vocal music group, χ^2^, chi‐squared test.

^a^ Likert scale 0–5 (0 = never, 1 = rarely, 2 = once a month, 3 = once a week, 4 = two to three times a week, and 5 = daily).

^b^ Classification based on BDAE/ASRS: scores 0–4 = aphasia, score 5 = no aphasia. For aphasic patients, the mean score is shown.

^c^ Classification based on the MBEA Scale and Rhythm subtest average score (<75% cut‐off).

**Table 3:** Cognitive performance of the patients at different time points.

	Stage	VMG *N* = 27/26	IMG *N* = 23	ABG *N* = 33/32	*P*‐value
Verbal memory (score range 0–124)	T0	43.3 (22.6)	52.3 (14.8)	51.3 (21.8)	0.211
	T1	65.3 (20.7)	62.0 (17.9)	64.3 (21.2)	**0.002**
	T2	63.4 (21.3)	66.8 (17.4)	63.2 (18.9)	**0.002**
Language skills (score range 0–80)	T0	48.7 (19.2)	56.8 (6.6)	54.2 (13.1)	0.431
	T1	60.1 (10.0)	65.8 (6.3)	59.4 (12.2)	**0.009**
	T2	58.5 (8.0)	63.8 (4.3)	61.3 (12.0)	0.297
Focused attention: correct responses (score range 0–90)	T0	75.6 (20.4)	76.0 (20.1)	76.9 (17.9)	0.953
	T1	85.3 (11.2)	87.4 (3.6)	84.1 (11.5)	0.303
	T2	88.4 (1.8)	88.5 (2.4)	85.0 (7.8)	0.303
Focused attention: reaction times (s)	T0	3.8 (1.9)	5.1 (6.0)	4.6 (2.5)	0.189
	T1	2.7 (1.0)	4.2 (5.2)	3.0 (1.4)	0.863
	T2	2.7 (1.4)	4.5 (6.1)	2.9 (1.3)	0.717

Data are mean (SD). Higher scores indicate better outcome. Significant group differences are shown in bold. Abbreviations: ABG, Audiobook group; IMG, Instrumental music group; T0, baseline (acute); T1, 3‐month stage; T2, 6‐month stage; VMG, Vocal music group. The *P*‐values are from Group main effect in univariate ANOVA at T0 and from Time × Group interaction in mixed‐model ANOVA at T1 (T0 vs. T1) and T2 (T0 vs. T2).

**Table 4:** Mood of the patients at different time points.

	Stage	VMG *N* = 27/26	IMG *N* = 23	ABG *N* = 33/32	*P*‐value
POMS Tension (score range 0–16)	T0	3.8 (3.2)	4.4 (3.2)	4.2 (3.6)	0.684
	T1	3.9 (3.3)	2.9 (2.2)	3.0 (2.8)	0.597
	T2	3.5 (3.4)	2.6 (2.0)	3.3 (2.8)	0.875
POMS Depression (score range 0–28)	T0	5.3 (5.7)	7.0 (6.3)	6.7 (6.9)	0.560
	T1	3.8 (3.9)	3.0 (2.6)	5.7 (5.4)	0.103
	T2	4.9 (5.6)	3.5 (3.0)	5.1 (5.9)	0.612
POMS Irritability (score range 0–28)	T0	4.1 (5.6)	6.1 (4.6)	4.4 (6.0)	0.753
	T1	5.8 (4.4)	3.4 (3.5)	5.4 (6.9)	0.164
	T2	5.9 (5.5)	2.9 (3.1)	5.2 (5.2)	0.401
POMS Vigor (score range 0–24)	T0	10.9 (5.5)	8.1 (5.1)	8.8 (5.2)	0.152
	T1	13.7 (6.9)	12.0 (6.4)	11.3 (5.9)	0.387
	T2	13.0 (6.6)	13.3 (5.6)	11.6 (6.3)	0.522
POMS Fatigue (score range 0–12)	T0	5.5 (2.7)	5.5 (2.8)	5.0 (2.9)	0.822
	T1	4.1 (2.3)	4.0 (2.7)	4.7 (3.1)	0.913
	T2	4.1 (3.0)	3.3 (2.9)	3.5 (3.1)	0.567
POMS Inertia (score range 0–12)	T0	2.6 (2.0)	3.0 (2.5)	3.0 (2.9)	0.813
	T1	2.6 (2.7)	2.3 (2.4)	3.2 (2.4)	0.639
	T2	2.8 (2.7)	2.6 (2.9)	2.9 (3.1)	0.405
POMS Confusion (score range 0–20)	T0	7.4 (4.3)	7.2 (4.6)	7.4 (4.5)	0.884
	T1	3.5 (3.7)	2.4 (2.8)	5.1 (3.4)	0.096
	T2	3.8 (3.7)	2.8 (2.8)	3.5 (3.2)	0.992
POMS Forgetfulness (score range 0–12)	T0	4.2 (2.9)	4.1 (2.6)	4.2 (2.5)	0.796
	T1	3.4 (2.6)	3.0 (2.6)	3.5 (2.4)	0.768
	T2	3.4 (3.0)	3.0 (3.0)	3.2 (2.7)	0.813

Data are mean (SD). Higher scores indicate better outcome. Significant group differences are shown in bold. Abbreviations: ABG, Audiobook group; IMG, Instrumental music group; T0, baseline (acute); T1, 3‐month stage; T2, 6‐month stage; VMG, Vocal music group. The *P*‐values are from Group main effect in univariate ANOVA at T0 and from Time × Group interaction in mixed‐model ANOVA at T1 (T0 vs. T1) and T2 (T0 vs. T2).

### Group comparability during the follow‐up

The amount of motor, speech, or cognitive rehabilitation received by the patients at T1 and T2 was comparable between the groups (Table 5). The frequency of music and audiobook listening differed highly significantly between the groups, both during the intervention and follow‐up periods. Listening frequency was higher for music in VMG and IMG than in ABG, and for audiobooks in ABG than in VMG and IMG at T1 (*P* < 0.001 in all) and, to a lesser extent, at T2 (*P* < 0.092 in all). There were no significant differences in music or audiobook listening between VMG and IMG. The average amount of daily listening to the allocated material was 1.8 h (SD = 0.9), totaling around 100 h (*M* = 107.9, SD = 55.1) over the 2‐month intervention period. Even though at group level the listening frequencies followed the study protocol, there was a significant difference between the groups in cross‐listening (using own devices to listen to material not part of the protocol: music in ABG, audiobooks in VMG and IMG), indicative of treatment contamination, during the intervention period (*H* = 42.64, *P* < 0.001), with ABG showing more cross‐listening than VMG and IMG (*P* < 0.001 in both).

**Table 5:** Listening activity and received rehabilitation of patients during the follow‐up.

	VMG *N* = 27/26	IMG *N* = 23	ABG *N* = 33/32	*P*‐value
Listening and rehabilitation at T1				
Listening to music^a^	5.0 (0.0)	5.0 (0.0)	2.7 (2.1)	**<0.001 (H)**
Listening to audiobooks^a^	0.0 (0.2)	0.1 (0.7)	4.6 (1.0)	**<0.001 (H)**
Cross‐listening of material^a^	0.0 (0.2)	0.1 (0.7)	2.7 (2.1)	**<0.001 (H)**
Received motor rehabilitation^b^	28.6 (34.3)	15.7 (28.0)	15.5 (21.5)	0.180 (H)
Received speech rehabilitation^b^	4.8 (7.8)	2.9 (5.4)	3.8 (7.0)	0.804 (H)
Received cognitive rehabilitation^b^	5.6 (12.8)	1.7 (3.7)	1.5 (3.1)	0.342 (H)
Listening and rehabilitation at T2				
Listening to music^a^	4.2 (1.2)	3.7 (1.5)	2.9 (1.6)	**0.008 (H)**
Listening to audiobooks^a^	0.2 (0.8)	0.4 (1.1)	2.8 (2.0)	**<0.001 (H)**
Received motor rehabilitation^b^	39.6 (44.8)	24.8 (42.8)	27.5 (39.6)	0.282 (H)
Received speech rehabilitation^b^	6.2 (12.7)	4.2 (9.7)	4.6 (7.5)	0.764 (H)
Received cognitive rehabilitation^b^	7.1 (13.4)	2.9 (5.3)	4.4 (7.4)	0.559 (H)

Data are mean (SD). Significant group differences are shown in bold. Abbreviations: ABG, Audiobook group; H, Kruskal–Wallis test; IMG, Instrumental music group; T0, baseline (acute); VMG, Vocal music group.

^a^ Likert scale 0–5 (0 = never, 1 = rarely, 2 = once a month, 3 = once a week, 4 = two to three times a week, 5 = daily).

^b^ Number of therapy sessions (motor: physical or occupational therapy, speech: speech therapy, cognitive: neuropsychological rehabilitation).

### Effects of music listening on behavioral recovery

**Figure 2:**
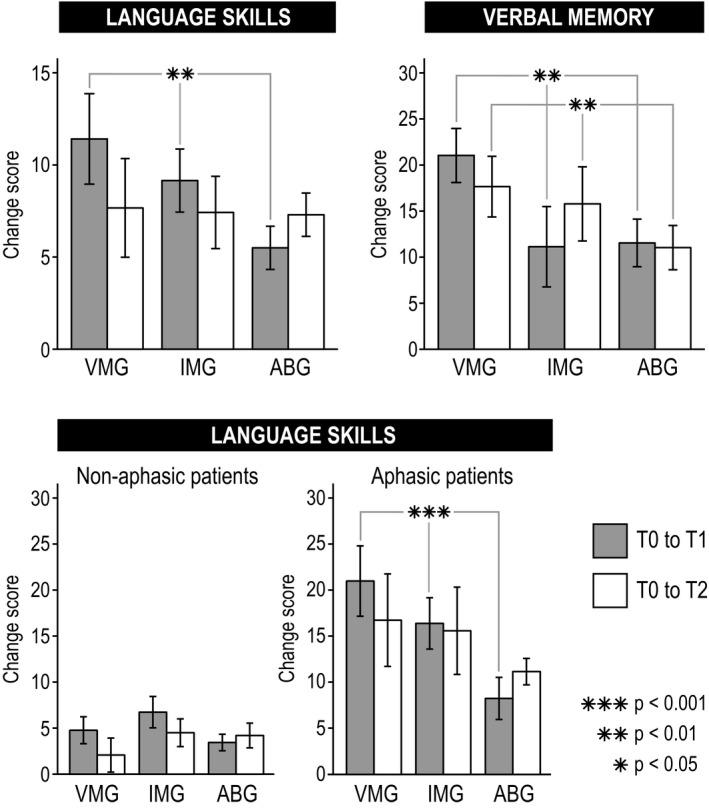
Significant changes in language skills and verbal memory and (mean ± SEM) from acute to 3‐month (gray bars) and 6‐month (white bars) poststroke stage are shown in the three groups. Upper panel shows results across all patients and lower panel within non‐aphasic and aphasic patients. The significance of the Time × Group interaction is shown with asterisks. ABG = Audiobook group, IMG = Instrumental music group, T0 = baseline (acute), T1 = 3‐month stage, T2 = 6‐month stage, VMG = Vocal music group.

Longitudinal results of the cognitive outcome measures are shown in Figure 2, Table 3, and Figure S1. In the primary outcome measure (verbal memory), there were significant Time × Group interactions from T0 to T1 [*F*(2,67) = 6.96, *P* = 0.002, ηp^2^ = 0.172] and from T0 to T2 [*F*(2,66) = 7.10, *P* = 0.002, ηp^2^ = 0.177]. Post hoc testing (Bonferroni‐corrected) showed that verbal memory improved more in VMG than in IMG from T0 to T1 (*P* = 0.015) and more in VMG than in ABG from T0 to T2 (*P = *0.046). In the secondary outcome measures, language showed significant Time × Group [*F*(2,58) = 5.12, *P* = 0.009, ηp^2^ = 0.150] and Time × Group × Aphasia [*F*(2,58) = 3.42, *P* = 0.039, ηp^2^ = 0.105] interactions from T0 to T1. A post hoc subgroup analysis with separate mixed‐model ANOVAs within aphasics and non‐aphasics showed that the Time × Group interaction was significant only in the aphasics [*F*(2,18) = 12.82, *P* < 0.001, ηp^2^ = 0.588]. Post hoc testing (Bonferroni‐corrected) within the aphasics showed that language recovery was better in VMG than in ABG (*P* = 0.002). There were no significant effects in focused attention (hits and RTs) or in the POMS scales (see Table 4 and Fig. S2).

### Effects of music listening on structural neuroplasticity

**Figure 3:**
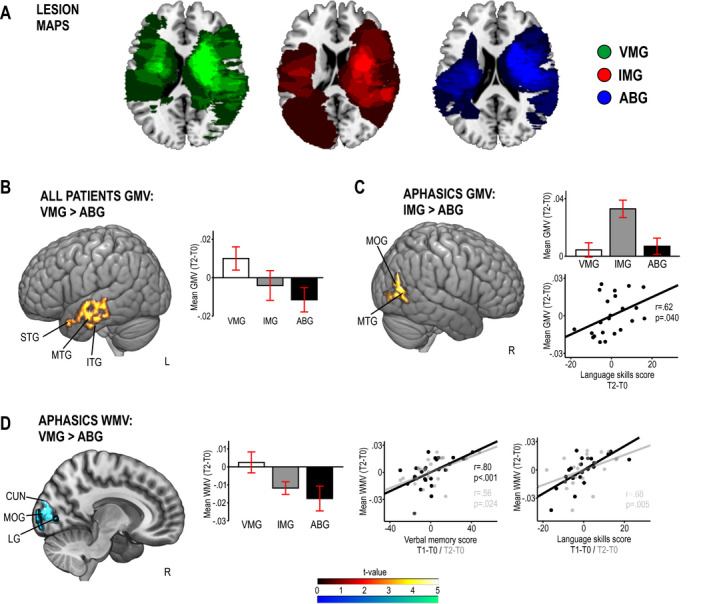
Pooled voxel‐based morphometry (VBM) results from the Helsinki and Turku studies (*N* = 75). Panel A: Lesion overlay maps of the three groups. Panels: B‐D: Significant group differences in VBM from acute (T0) to 6‐month (T2) stage in (B) gray matter volume (GMV) across all patients, (C) GMV within aphasic patients, and (D) white matter volume (WMV) within aphasic patients. Data reported in the histograms are mean ± SEM. Correlations to change in language/verbal memory are shown with scatter plots. Results are at *P* < 0.005 (uncorrected) and only clusters surviving a FWE‐corrected *P* < 0.05 threshold are shown and labeled. ABG = Audiobook group, IMG = Instrumental music group, T0 = baseline (acute), T1 = 3‐month stage, T2 = 6‐month stage, VMG = Vocal music group. Anatomical abbreviations: CUN = cuneus, ITG = inferior temporal gyrus, LG = lingual gyrus, MOG = middle occipital gyrus, MTG = middle temporal gyrus, STG = superior temporal gyrus.

In the longitudinal VBM analyses (secondary outcome, see Fig. 3 and Table 6), GMV increased more in VMG than in ABG in one cluster in left temporal [superior (STG), middle (MTG), and inferior (ITG) temporal gyrus] areas across all patients from T0 to T2 (Fig. 3B). In aphasics, WMV increased more in VMG than in ABG in one cluster comprising right medial parieto‐occipital [lingual gyrus (LG), cuneus, middle occipital gyrus (MOG)] areas (Fig. 3D), and the increased WMV in this cluster correlated with the improvement in language and verbal memory from T0 to T1 (*r* = 0.72, *P* = 0.004 and *r* = 0.80, *P* < 0.001) and T2 (*r* = 0.68, *P* = 0.005 and *r* = 0.56, *P* = 0.024). In aphasics, there was also larger GMV increase in the IMG than in the ABG in one cluster in right temporoparietal (MTG, MOG) areas (Fig. 3C), and the increased GMV in this cluster correlated with the improvement in language from T0 to T2 (*r* = 0.64, *P* = 0.040).

**Table 6:** Gray and white matter volume changes (acute to 6‐month stage) in VBM analyses.

Patients	Group contrast	Volume type	Area	MNI coordinates	Cluster size	FWEc	t‐value	Correlation between volume and cognitive change			
								T1‐T0		T2‐T0	
								LAN	MEM	LAN	MEM
All	VMG > ABG	GMV	Left superior temporal gyrus (BA 22, 38)	−57 −7 −5	7070	7070	4.33**	n.s.	n.s.	n.s.	n.s.
			Left middle temporal gyrus (BA 21)	−62 −16 −1							
			Left inferior temporal gyrus (BA 20)	−62 −10 −18							
Aphasics	VMG > ABG	WMV	Right lingual gyrus	10 −79 0	2498	2498	6.17*	*r* = 0.80 *P* < 0.001	*r* = 0.56 *P* = 0.024	*r* = 0.72 *P* = 0.004	*r* = 0.68 *P* = 0.005
			Right cuneus	8 −87 9							
			Right middle occipital gyrus	9 −96 12							
	IMG > ABG	GMV	Right middle temporal gyrus (BA 37, 39)	44 −74 21	2893	2893	6.16*	n.s.	n.s.	n.s.	*r* = 0.62 *P* = 0.040
			Right middle occipital gyrus (BA 19)	47 −82 4							

All results are thresholded at a whole‐brain uncorrected *P* < 0.005 threshold at voxel level.

FWEc is the minimum number of voxels for a cluster to be significant at the FWE‐corrected *P* < 0.05 level, according to SPM standard cluster‐level correction based on random field theory and cluster‐forming threshold of *P* < 0.005.

ABG, Audiobook group; BA, Brodmann area; GMV, gray matter volume; IMG, Instrumental music group; LAN, language skills; MEM, verbal memory; VMG, Vocal music group; WMV, white matter volume.

* *P* < 0.05 FWE‐corrected at the cluster level.

** *P* < 0.005 FWE‐corrected at the cluster level.

### Effects of music listening on functional connectivity

**Figure 4:**
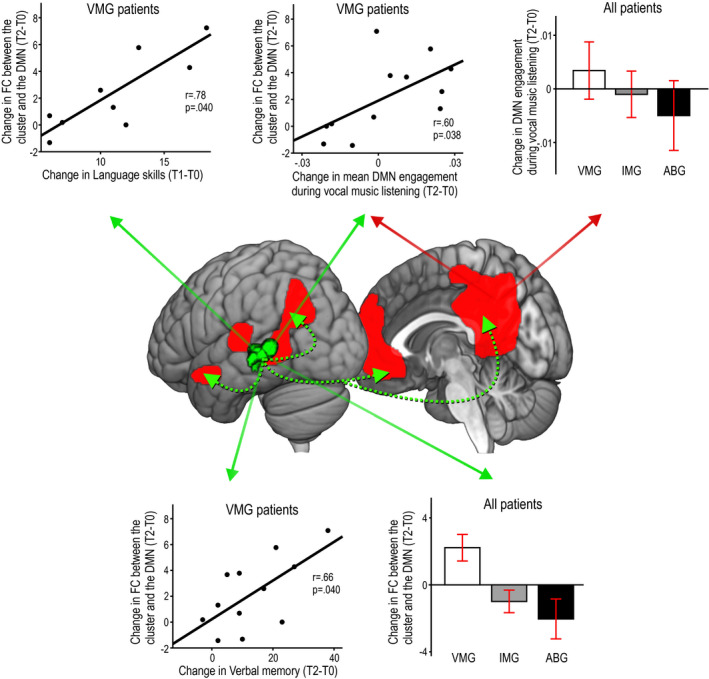
fMRI results on functional connectivity from the Turku study (*N* = 35). Significant group differences in functional connectivity (FC) from acute (T0) to 6‐month (T2) stage between the default mode network (DMN) and the left temporal (STG/MTG) areas in the resting‐state condition (cluster shown in green–black color scale) and the mean engagement of the DMN during the Vocal music listening condition (DMN illustrated in red color). Spatial results in the resting‐state condition are at *P* < 0.005 (uncorrected) and only the cluster surviving a FWE‐corrected *P* < 0.05 threshold is shown. Group differences are shown in histograms (mean ± SEM), and correlations within the VMG (*N* = 12) are shown in scatterplots. The histograms/scatterplots showing results in the resting‐state condition are marked with green solid arrows and those showing results in the vocal music listening condition are marked with red solid arrows. Dashed green arrows illustrate FC between the left temporal cluster and the other parts of the DMN. ABG = Audiobook group, IMG = Instrumental music group, T0 = baseline (acute), T1 = 3‐month stage, T2 = 6‐month stage, VMG = Vocal music group.

Given that the music‐induced GMV/WMV changes were located primarily within the posterior and temporal parts of the DMN,^23^ which has been linked also to episodic or verbal memory,^32^ we sought to determine as a further secondary outcome if FC changes in the DMN could underlie the cognitive benefits and structural neuroplasticity induced by the vocal music listening (Fig. 4 and Table 7). In the rs‐fMRI ICA analysis, which focused on the spatial component of each brain network, VMG showed a larger increase in FC between left temporal (STG, MTG) areas and the rest of the DMN than ABG or IMG from T0 to T2. Moreover, VMG also showed increased FC between right temporal [STG, Heschl's gyrus (HG)] areas and the rest of the DMN than IMG from T0 to T2. In the task‐fMRI, there was a significant Time × Group interaction from T0 to T2 [*F*(1,25) = 3.73, *P* = 0.038] in whole network‐level DMN engagement in the Vocal condition, with post hoc tests (Bonferroni‐corrected) showing a larger increase in VMG than in ABG (*P* = 0.041). No significant effects were observed in the Instrumental and Speech conditions. Correlation analyses showed that in VMG patients, the increased resting‐state FC between the different clusters of the DMN and the left STG/MTG correlated with the improvement in language (T0 to T1: *r* = 0.78, *P* = 0.040) and verbal memory (T0 to T2: *r* = 0.66, *P* = 0.040). Interestingly, this increase in rs‐fMRI connectivity between left temporal regions and the DMN also correlated with the mean DMN engagement during the Vocal condition: the greater the DMN engagement after 6 months while listening to vocal music, the more functionally connected the left temporal lobe was with the DMN (*r* = 0.60, *P* = 0.038).

**Table 7:** Functional connectivity changes (acute to 6‐month stage) in fMRI analyses.

Group contrast	Area	MNI coordinates	Cluster size	FWEc	t‐value
VMG > ABG	Left superior temporal gyrus	−44 −34 4	342	342	4.52*
	Left middle temporal gyrus	−56 to−40 4			
VMG > IMG	Left middle temporal gyrus	−46 −48 3	338	296	4.98*
	Left superior temporal gyrus	−45 −31 2			
	Right Heschl's gyrus	46 −22 5	296		4.48*
	Right superior temporal gyrus	48 −18 4			

All results are thresholded at a whole‐brain uncorrected *P* < 0.005 threshold at voxel level.

FWEc is the minimum number of voxels for a cluster to be significant at the FWE‐corrected *P* < 0.05 level, according to SPM standard cluster‐level correction based on random field theory and cluster‐forming threshold of *P* < 0.005.

ABG, Audiobook group; IMG, Instrumental music group; VMG, Vocal music group.

* *P* < 0.05 FWE‐corrected at the cluster level.

## Discussion

The present study set out to verify and extend previous results on beneficial effects of daily music listening on cognitive, emotional, and neural recovery after stroke^9^, ^10^, ^11^ and to explore whether the vocal (sung) component of music plays a key role in its rehabilitative efficacy. Specifically, we pooled data from two single‐blind parallel‐group RCTs of stroke patients (total *N* = 83), comprising our previous trial^9^, ^10^ (*N* = 38) and a new trial (*N* = 45), which both had a 6‐month follow‐up and utilized a combination of cognitive, emotional, and neuroimaging outcome measures. Our main findings were that compared to audiobooks vocal music listening enhanced the recovery of verbal memory and language. An exploratory post hoc subgroup analysis suggested that especially aphasic patients may benefit from listening to vocal music as a means to facilitate early language recovery. These positive effects of vocal music listening were coupled with increased structural neuroplasticity, indicated by GMV/WMV changes, in left temporal areas in all patients and in right medial parietal areas in aphasics and with functional neuroplasticity, indicated by resting‐state and task‐related FC increases in the DMN.

The specific enhancement of language and verbal memory induced by vocal music suggests that the sung (vocal) component of music listening is crucial for its rehabilitative effect. Conceptually, songs represent an interface between speech and music, binding together lyrics and melody and providing a structured temporal scaffolding framework that facilitates their recall. The close coupling of vocal music and verbal memory is evidenced by behavioral studies of stroke patients showing that (1) verbal material (stories) is learned and recalled better when presented in sung than spoken format^33^ and (2) overt production of verbal material during memory encoding is more effective for later recall when done through singing than speaking.^20^ Even though VMG patients were not instructed to sing along to the songs, it is plausible that listening to the songs may have elicited subvocal processing, which could have covertly trained the phonological loop function of working memory, leading to an enhanced verbal memory recovery. Subvocal training could also underlie the enhancement of language skills induced by the vocal music listening, especially in aphasic patients, as singing‐based rehabilitation has been found effective for speech in aphasia.^21^, ^22^

Vocal music can also enhance vigilance or arousal,^34^ which is likely mediated by emotional factors. Music evokes strong emotions and induces pleasure and rewarding experiences which arise from increased activation of the mesolimbic reward system^4^, ^35^ in which dopamine plays a causal role.^36^ Given that music‐induced pleasure and engagement of the limbic network are higher when listening to familiar versus unfamiliar music^37^ and sung versus instrumental music^15^, ^16^ and that the individual reward value of music mediates its positive effect on episodic memory,^38^ it is possible that the observed positive effect of vocal music on verbal memory recovery is at least partly driven by its intrinsic ability to engage motivation‐ and reward‐related dopaminergic networks.

Although music listening has a general mood‐enhancing and stress‐reducing effect in daily life,^39^ the effects of music listening on poststroke mood, as measured by POMS, were not significant in the present study when compared to audiobook listening (as shown in Table 4, there was a slight trend for reduced Depression and Confusion in the music groups compared to the ABG at T1, but the group effect did not reach significance). Given also our previous results where the positive effects of music listening on POMS Depression and Confusion were seen only when compared to the standard care control group^9^ as well as the results of Baylan et al.^11^ where the effects of music and audiobook listening did not differ on another clinical mood scale (Hospital Anxiety and Depression Scale), the impact of daily music listening for enhancing mood after stroke is still unclear, at least when compared to another stimulating recreational activity.

Our previous exploratory VBM findings indicated that in left hemisphere‐lesioned stroke patients (*N* = 23), music listening increased GMV in left and right superior frontal gyrus, left anterior cingulate, and right ventral striatum compared to audiobooks and standard care.^10^ Using a larger sample (*N* = 75) and more rigorous statistical criteria (FWE‐correction), the present results showed that compared to audiobooks, vocal music listening specifically increased GMV in left temporal areas (STG, MTG, ITG) areas across all patients. Stronger activation in left temporal regions has been reported in previous fMRI studies of healthy subjects comparing song and speech listening.^13^, ^14^ These regions also play a crucial role in perceiving the spectrotemporal structure of sounds^40^ as well as in the combinatorial processing of lexical, phonological, and articulatory features of speech^41^ and also in verbal working memory.^42^, ^43^ In aphasic patients, increased GMV/WMV induced by vocal or instrumental music listening was seen in right medial parieto‐occipital areas (LG, cuneus, MOG) and in posterior temporal (MTG) areas, which have been linked to music and speech perception^44^ and memory‐related visual imagery.^45^ Importantly, the volume changes in these posterior temporal and parietal regions correlated with enhanced recovery of language and verbal memory, which is in line with previously reported therapy‐induced changes in aphasia in these regions.^46^ Notably, the VMG > ABG effects were partly driven by a reduction of GMV in ABG in these clusters. After stroke, spared brain regions can show both volume increase, which indicates recovery‐related neuroplasticity, and volume decrease, which indicates atrophy and is associated with poor recovery and lower gains induced by rehabilitation.^47^ It is possible that the large‐scale neural activation induced by music listening after stroke^17^ can have a long‐term neuroprotective impact by preventing atrophy in cortical areas most strongly activated by songs.

Music listening induced also long‐term FC changes in the DMN. As the DNM is linked to emotional processing, self‐referential mental activity, and the recollection of prior experiences,^23^ it is strongly engaged also when listening to music, especially when it is familiar and self‐preferred.^48^ In line with this, VMG showed larger increase in FC in the whole DMN during vocal music (but not instrumental music or speech) listening than ABG, indicating functional neuroplasticity specific to the type of stimulus and intervention. Importantly, also resting‐state FC in the left temporal (STG/MTG) areas of the DMN increased more in VMG than in ABG or IMG, and correlated with the improved recovery of language and verbal memory. Previously, reduced DMN connectivity has been associated with verbal memory impairment in aging^32^ and after stroke^49^ and increased DMN connectivity with successful stroke recovery.^25^ Together, the VBM and FC results provide compelling evidence that the rehabilitative effect of vocal music is underpinned by both structural and functional plasticity changes in temporoparietal networks crucial for emotional processing, language, and memory.

The present study has some methodological limitations, which should be taken into account when evaluating its findings. First of all, the study was not a single RCT but a pooled analysis of two RCTs, one with randomization to three (VMG/IMG/ABG) groups (Turku) and the other with randomization to two groups (MG/ABG) and then post hoc reclassification to three (VMG/IMG/ABG) groups (Helsinki). The results may therefore include a slight self‐selection bias, more so between the two music groups and less so between the music groups and the ABG. However, given that the study design of the two trials was otherwise highly similar and that all outcome measure results were covaried for trial site, we do not feel that this represents a significant bias. Regarding power, due to the combined sample size of the two studies, the pooled analysis had greater test power than each of the individual studies. Using G*Power, we performed a post hoc calculation of the achieved power based on the effect sizes of the original Helsinki study.^9^ This showed that the pooled sample yielded 97% power for the primary outcome (verbal memory) and 76%–87% power for the secondary outcomes (language skills, focused attention, POMS Depression, POMS Confusion) to detect a significant change between groups from T0 to T1, suggesting that the study was sufficiently powered. The effect sizes (ηp^2^) in the present study for the efficacy of music listening on verbal memory and language recovery were of medium level, reaching a large level for the efficacy of vocal music listening on language recovery in aphasics. Second, owing to the relatively small sample sizes in the subgroup analyses of aphasic patients (*N* = 29) and in the fMRI analyses (*N* = 35), their results should be considered still somewhat tentative and need to be confirmed with larger studies. Especially studies of aphasic patients with more varying severity levels and aphasia subtypes are warranted. Also uncovering if and how different demographic and clinical background factors, such as prestroke music listening and amusia which were included as covariates in the analyses, mediate the efficacy of music listening need to be explored in a larger trial, as this could pave the way toward more individualized use of music listening in stroke rehabilitation.

Clinically, the findings address a vital issue of how the patient environment can be optimized for recovery during the first weeks after stroke when typically over 70% of daily time is spent in nontherapeutic activities^50^ even though this time‐window is ideal for rehabilitation from the standpoint of neuroplasticity. Corroborating previous findings,^9^, ^10^, ^11^ the present study provides further evidence for the use of music listening as an effective, easily applicable, and inexpensive way to support cognitive recovery after stroke. Importantly, our results show for the first time that the vocal (sung) component of music is driving its rehabilitative effect on verbal memory and that vocal music can also speed up language recovery in aphasia during the first 3 months. The reason why the positive effect of vocal music occurs particularly at the early poststroke stage is likely linked to the dynamic pattern of language reorganization in aphasia, where upregulation of both left and right frontotemporal regions takes place at the early (first weeks and months) recovery stage, followed by more pronounced reorganization of perilesional left regions at the chronic (6‐month) stage.^18^ It is plausible that vocal music engages and stimulates the bilateral frontotemporal network more extensively than audiobooks, leading to a better language recovery in aphasia at the early stage, whereas the effects begin to level off at the chronic stage when left hemisphere mechanisms (engaged more evenly by vocal music and audiobooks) become more dominant. Although more research is still needed to verify the effects of vocal music listening on aphasia, our novel findings suggest that it could perhaps be used to supplement speech therapy, which is often difficult to implement at the early poststroke stage due to severity of symptoms, general fatigue, and lack of rehabilitation resources.

## Author Contributions

A.J.S., V.L., A.R.‐F., M.T., S.S., and T.S. designed the study protocol. A.J.S., Pa.R., J.S., T.A., A.R.‐F., and T.S. designed the s/fMRI measures, and V.L., M.L., and T.S. designed the neuropsychological measures of the study. T.L., A.Y., Pe.R., S.L., and A.F. implemented the intervention. A.J.S., V.L., Pa.R., and T.S. collected and/or analyzed the data. A.J.S., V.L., and T.S. wrote the manuscript, with help from all the authors on the interpretation and discussion of the results.

## Conflict of Interest

Nothing to report.

## Supporting information


**Figure S1.** Bar charts displaying the cognitive domain scores (mean ± SD) of the patients at the acute (T0), 3‐month (T1), and 6‐month (T2) poststroke stages.

**Figure S2.** Bar charts displaying the Profile of Mood States (POMS) scale scores (mean ± SD) of the patients at the acute (T0), 3‐month (T1), and 6‐month (T2) poststroke stages.
