# Supplementary material for: Vocal music enhances memory and language recovery after stroke: pooled results from two RCTs
Source: Ann Clin Transl Neurol. 2020 Oct 6;7(11):2272–87. doi: 10.1002/acn3.51217 (PMC7664275; doi:10.1002/acn3.51217)
Supplement: Supplementary file 1 — Figure S1. Bar charts displaying the cognitive domain scores (mean ± SD) of the patients at the acute (T0), 3‐month (T1), and 6‐month (T2) poststroke stages. [file ACN3-7-2272-s001.pdf]

Sihvonen et al.
Vocal Music Enhances Memory and Language Recovery after Stroke: Pooled Results from Two RCTs
Annals of Clinical and Translational Neurology

Supplementary Material / Supplementary Figure 1

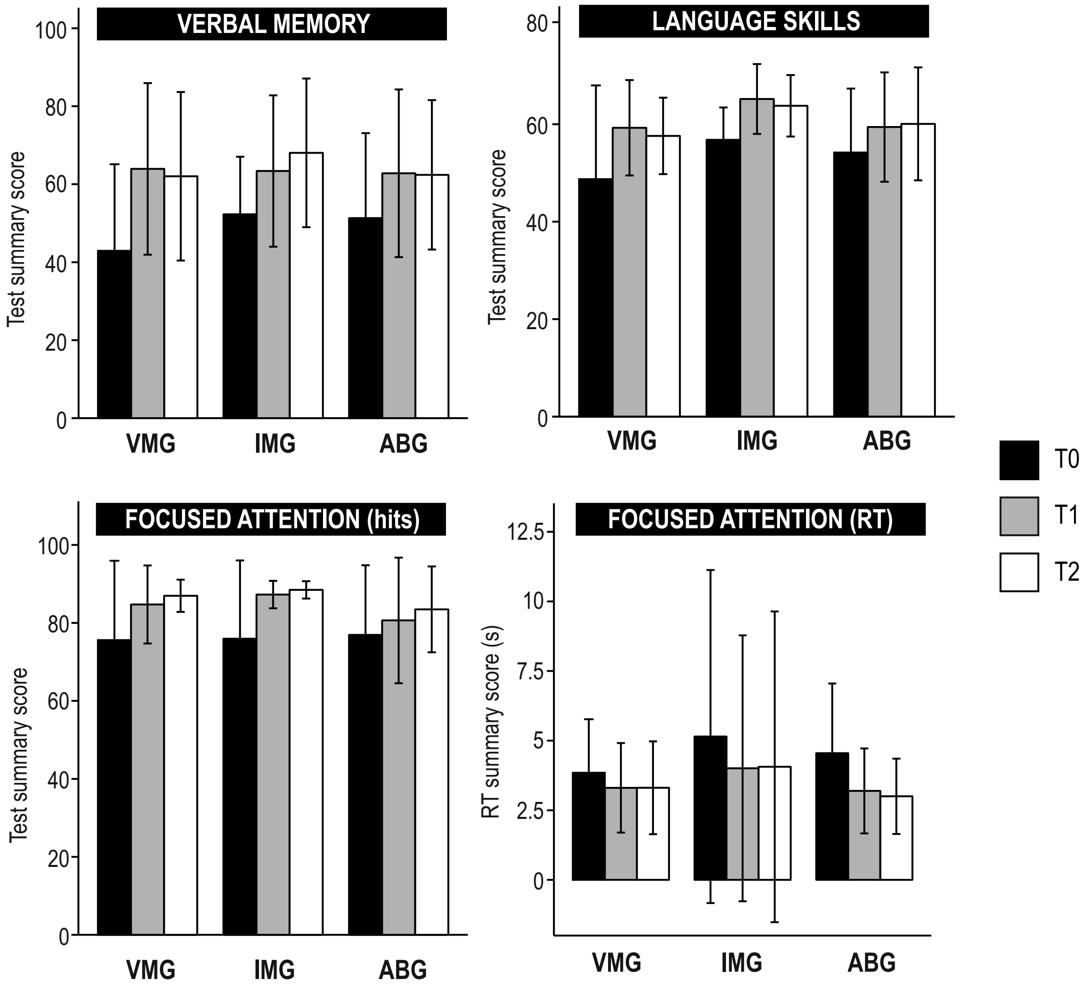

Supplementary Figure 1. Bar charts displaying the cognitive domain scores (mean ± SD) of the patients at the acute (T0), 3-month (T1) and 6-month (T2) post-stroke stages. ABG = Audio book group, IMG = Instrumental music group, RT = reaction time, VMG = Vocal music group.
